# Wavelet-enhanced spatiotemporal connectivity-preserving network for intracranial artery segmentation in DSA sequences

**DOI:** 10.1038/s41598-026-51440-y

**Published:** 2026-05-19

**Authors:** Yingchao He, Mingfeng Lv, Yi Yang, Yiya Xu, Zhiwei Song, Xiaolin Jiang, Yinzhou Wang

**Affiliations:** 1 Department of Neurology, Fuzhou University Affiliated Provincial Hospital, Fuzhou, 350000 Fujian China; 2 Xiamen University, No. 4221, Xiang’an South Road,Xiang’an District, Xiamen, 361000 Fujian China; 3 Shengli Clinical Medical College of Fujian Medical University, No. 88, Traffic Road, Gulou District, Fuzhou, 350000 Fujian China; 4 Fujian Key Laboratory of Medical Analysis, Fujian Academy of Medical Sciences, No. 7, Wusi Road, Gulou District, Fuzhou, 350000 Fujian China

**Keywords:** Digital subtraction angiography, Cerebrovascular segmentation, Spatiotemporal learning, Computational biology and bioinformatics, Engineering, Mathematics and computing, Neurology, Neuroscience

## Abstract

Accurate intracranial artery segmentation from Digital Subtraction Angiography is critical for the diagnosis and interventional treatment of cerebrovascular diseases. However, traditional methods struggle to capture dynamic spatiotemporal dependencies, often leading to vascular discontinuity and the loss of fine distal vessels due to background interference and resolution loss. In this study, we propose the Wavelet-Enhanced Spatiotemporal Connectivity-Preserving Network (WESCP-Net), a novel framework designed to synergize physical priors with frequency-domain feature extraction. Specifically, we introduce a Physically-Guided Spatiotemporal Enhancement module that explicitly exploits hemodynamic flow variance to differentiate active vascular signals from static artifacts. To address the loss of high-frequency spatial details in standard downsampling, we incorporate a Wavelet-Integrated Encoder and a Topology-Aware Reconstruction module, which utilize discrete wavelet transforms to preserve sharp vessel boundaries and restore structural connectivity. Experimental results on the DIAS dataset demonstrate that WESCP-Net achieves state-of-the-art performance, yielding a Dice Similarity Coefficient of 0.7982 and an Intersection over Union score of 0.6422. Notably, its connectivity-preserving mechanism achieves a clDice metric of 0.7135, improving the continuity of vascular terminals. WESCP-Net provides a robust technological paradigm for precise cerebrovascular segmentation, facilitating reliable surgical navigation and quantitative diagnosis.

## Introduction

Cerebrovascular diseases, particularly stroke, represent a critical global health challenge, ranking as a leading cause of mortality and long-term disability worldwide^1^. The sudden onset of neurological deficits necessitates rapid and precise diagnosis to inform intervention strategies. Among various imaging modalities, Digital Subtraction Angiography (DSA) is regarded as the “gold standard” for diagnosing intracranial vascular pathologies due to its superior spatial resolution and dynamic visualization of blood flow. Unlike static modalities such as Magnetic Resonance Angiography (MRA) or Computed Tomography Angiography (CTA), DSA provides a 2D+time spatiotemporal sequence, offering hemodynamic information essential for preoperative planning and intraoperative navigation.

However, the automated segmentation of cerebral vasculature from DSA sequences presents unique challenges that generic deep learning models struggle to address. The primary difficulty stems from the inherent conflict between the spatiotemporal complexity of the data and the architectural limitations of standard Convolutional Neural Networks (CNNs). DSA sequences characterize the temporal progression of contrast agent flow, where treating frames as independent static images may overlook relevant hemodynamic continuity. Intracranial arteries exhibit intricate topologies with varying calibers. Standard architectures like U-Net^2^ typically rely on pooling operations for downsampling, which essentially act as destructive low-pass filters. This process inevitably discards the high-frequency spatial details necessary for reconstructing fine distal vessels, leading to the “disconnected vessel” phenomenon. Compounding these structural challenges are patient motion and subtraction artifacts, which mimic vascular features and degrade the signal-to-noise ratio.

Deep learning has significantly advanced medical image analysis^3^, yet addressing specific challenges in DSA segmentation remains a complex task. Most approaches either process data frame-by-frame, ignoring temporal coherence, or employ computationally heavy 3D networks that struggle to handle the high spatial resolution of angiographic data effectively. To address these limitations, we propose the Wavelet-Enhanced Spatiotemporal Connectivity-Preserving Network (WESCP-Net). By integrating discrete wavelet theory into the deep learning backbone, our framework replaces destructive pooling with lossless frequency decomposition, ensuring that high-frequency vascular edges are preserved throughout the encoding process. Simultaneously, we incorporate physically-guided mechanisms to leverage the temporal variance of blood flow, allowing the model to distinguish true hemodynamic transients from static background noise.

Our main contributions are as follows:

- We introduce a Wavelet-Integrated Feature Encoding strategy. By embedding the Discrete Wavelet Transform (DWT) into the backbone, we address the inherent information loss in standard CNN downsampling, enabling the network to explicitly preserve high-frequency vascular boundaries.
- We propose the Physically-Guided Spatiotemporal Enhancement (PGSE) module. This unit dynamically captures hemodynamic flows across frames, allowing the network to distinguish active vasculature from static background artifacts based on physical temporal coherence.
- To resolve topological discontinuities, we propose a Topology-Aware Reconstruction mechanism. We adapt and enhance the Efficient Upsampling Convolution Block (EUCB) by integrating wavelet transforms and channel shuffles. This improved block recovers fine distal branches that are typically lost during reconstruction.
- Comprehensive evaluations on the DIAS dataset indicate that WESCP-Net achieves competitive performance compared to state-of-the-art methods, with notable improvements in topological connectivity (clDice) and segmentation accuracy.

## Related work

Cerebrovascular segmentation is important for the diagnosis, quantification, and treatment planning of cerebrovascular diseases. This section reviews existing datasets, methodological evolutions from traditional to deep learning approaches, and the clinical integration of these technologies.

### Datasets for cerebrovascular segmentation

High-quality datasets are the foundation of intelligent diagnosis. Common imaging modalities include MRA, CTA, and DSA. While non-invasive techniques like MRA are widely used for screening, DSA remains the gold standard for interventional guidance due to its high spatial resolution and dynamic flow visualization. Table 1 summarizes the datasets utilized in this field.

**Table 1:** Overview of commonly used cerebrovascular imaging datasets.

Dataset	Type	Numbers	Dimension
Public healthy MR	MR	100	3D
IXI	MR	600	3D
A-SegAN	MRA	45	3D
CAS 2023	MRA	150	3D
DIAS	DSA	120	2D + Time

In contrast to public MRA datasets like IXI, the DIAS dataset^4^ fills the gap for DSA imaging. Derived from ischemic stroke treatments, this dataset specifically targets DSA sequences, capturing the spatiotemporal dynamics essential for hemodynamic analysis.

### Methodological evolution in vascular segmentation

Deep learning has demonstrated superior robustness compared to traditional model-based or tracking-based methods^5,6^. The U-Net^2^ serves as the cornerstone of this field, with diverse variants emerging to enhance segmentation efficacy. To capture multi-scale vascular features more effectively, researchers have introduced volumetric adaptations like V-Net^7^, nested skip pathways in UNet++^8^, and residual connections in Res-UNet^9^. Furthermore, nnU-Net^10^ introduced a self-configuring framework with extensive data preprocessing, proving highly effective in general medical segmentation. To address the intricate structures of cerebral vessels, recent research has evaluated various U-Net families, emphasizing the importance of architectural depth for MRA segmentation^11^. Additionally, computationally efficient dilated residual networks have been proposed to expand the receptive field without increasing computational burden, facilitating rapid processing of vascular data^12^.

To further capture long-range dependencies and suppress noise, advanced mechanisms have been integrated. Transformer blocks have been extensively explored to augment global feature extraction capabilities, as demonstrated by architectures such as TransUNet^13^, Swin-UNet^14^, and UNETR^15^. Similarly, attention mechanisms have proven effective in low-contrast scenarios by highlighting salient regions. A multi-level residual dual attention network was specifically developed to recalibrate features adaptively, successfully segmenting major cerebral arteries by suppressing background artifacts^16^.

### Specific methods for DSA segmentation

While general vascular segmentation has advanced rapidly, research specifically on DSA presents unique challenges due to spatiotemporal redundancy and bone artifacts. Early attempts like DeepMedic were adapted for DSA vessel segmentation^17^. More recently, MDCNN^18^ leveraged multi-scale dense connections for single-frame DSA processing. To address topological breaks, ERNet^19^ proposed an edge regularization network utilizing erosion and dilation.

Recognizing the importance of temporal information, recent works have shifted towards sequence-based modeling. SVS-Net^20^ and VSS-Net^4^ introduced recurrent units and temporal attention to capture hemodynamic flows. However, a critical limitation of these methods is their reliance on standard convolutional downsampling, which inherently acts as a low-pass filter, leading to the loss of high-frequency details in thin distal vessels. They often lack explicit physical constraints to differentiate flow-related signals from static noise.

### Clinical integration and quantification

The ultimate goal of segmentation is to support clinical decision-making, such as aneurysm quantification or stenosis evaluation. Accurate quantification requires precise boundary delineation and noise reduction. Recent studies have highlighted the value of context-aware preprocessing to reduce the Volume of Interest (VOI), thereby enhancing the precision of downstream analysis^18,21^. Once segmented, these models facilitate automated volumetric quantification. For instance, deep learning approaches have been successfully applied to the automated segmentation of post-treatment intracranial aneurysms, enabling rigorous computer-aided volumetric quantification essential for evaluating treatment efficacy^22^. These advancements underscore the necessity for segmentation algorithms that are not only accurate at the pixel level but also topologically consistent for reliable clinical measurement^23–25^.

Despite the progress in static image segmentation, DSA is inherently spatiotemporal. Most existing methods treat DSA as independent frames or fail to fully exploit the frequency domain features. Our WESCP-Net addresses these gaps by introducing the Physically-Guided Spatiotemporal Enhancement (PGSE) and Wavelet-Integrated Feature Encoding, ensuring both topological continuity and effective noise suppression in the spatiotemporal domain.

## Method

In this section, we elaborate on the proposed WESCP-Net framework. We first present the overall architecture and the core spatiotemporal encoding mechanism, followed by detailed descriptions of the key components designed for physical enhancement and topology preservation.

### Network overview and sequence feature extraction module (SFEM)

**Figure 1:**
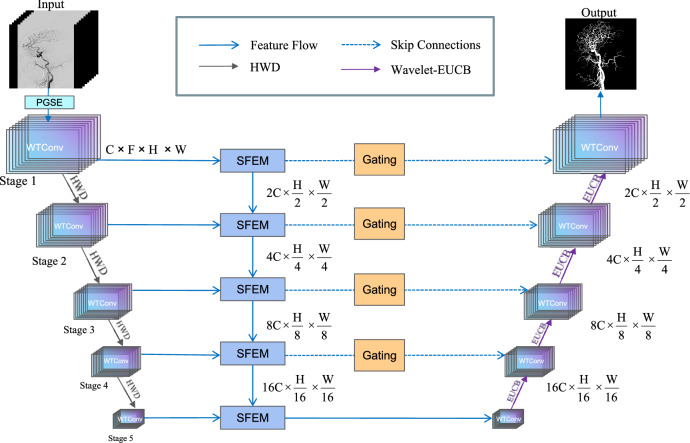
Architecture of the proposed WESCP-Net. The framework begins with the PGSE module for physically-guided signal enhancement. The encoder utilizes WTConv and HWD for frequency-aware feature extraction. A scale-adaptive injection strategy feeds the enhanced sequence into the Sequence Feature Extraction Module (SFEM) at multiple stages to preserve temporal details. Finally, the decoder employs Gating mechanisms and Wavelet-Enhanced EUCBs for topology-aware reconstruction.

The proposed WESCP-Net is conceptualized as a specialized spatiotemporal encoder-decoder framework designed to map high-dimensional DSA sequences into a precise 2D vascular segmentation mask. Built upon the VSS-Net baseline^4^, the architecture as illustrated in Fig. 1 integrates discrete wavelet theory with physically-guided enhancement mechanisms. Formally, the segmentation task is modeled as a dimensionality reduction problem^26^, where the input tensor $\textbf{S} \in \mathbb {R}^{T \times C \times H \times W}$ is projected onto a target space $\textbf{O} \in \mathbb {R}^{1 \times H \times W}$. To effectively capture the hemodynamic evolution inherent in 2D+t sequences while mitigating the loss of high-resolution temporal cues during deep feature encoding, we retain the Sequence Feature Extraction Module (SFEM) as the primary temporal encoding mechanism, specifically adapted to interact with our Wavelet-Based backbone.

A fundamental challenge in hierarchical encoding is the spatial resolution mismatch between the input sequence and the downsampled feature maps at deeper stages. The SFEM addresses this via a scale-adaptive injection strategy. Unlike standard architectures that ingest raw data only at the input layer, our SFEM re-injects the *physically-enhanced sequence* $\hat{\textbf{S}}$ (generated by the PGSE module, detailed in Section ''Physically-guided spatiotemporal enhancement (PGSE)'')) into the encoder’s deeper levels. Let $\textbf{X}_i \in \mathbb {R}^{T \times C_i \times H_i \times W_i}$ denote the feature map at the *i*th encoder stage. To incorporate the global temporal context, the SFEM first employs adaptive trilinear interpolation to strictly align the spatial dimensions of the enhanced source $\hat{\textbf{S}}$ with $\textbf{X}_i$. The aligned sequence $\tilde{\textbf{S}}_i$ is then concatenated with the feature map along the channel dimension. This fusion process supplements the deep semantic features with fine-grained texture information and is formulated as:

1 $$\begin{aligned} \tilde{\textbf{S}}_i = \mathscr {T}_{interp}(\hat{\textbf{S}}, (H_i, W_i)), \quad \textbf{Z}_i = \mathscr {F}_{fuse}([\textbf{X}_i, \tilde{\textbf{S}}_i]) \end{aligned}$$

where $\mathscr {T}_{interp}$ represents the interpolation function, $[\cdot , \cdot ]$ denotes channel-wise concatenation, and $\mathscr {F}_{fuse}$ is a *1 × 1* convolutional layer used to compress the channel dimensions of the hybrid feature $\textbf{Z}_i$.

To model the spatiotemporal dependencies within the fused features, we employ a Bi-directional Convolutional Gated Recurrent Unit (BiConvGRU)^27^. Unlike standard 2D convolutions that treat frames independently, the BiConvGRU captures the dynamic flow of contrast agents by propagating hidden states across the temporal dimension. The module processes the sequence in both forward and backward directions to leverage future context for resolving ambiguities in early wash-in phases. The hidden states $\textbf{H}_t^f$ (forward) and $\textbf{H}_t^b$ (backward) at time step *t* are updated as follows:

2 $$\begin{aligned} \textbf{H}_t^f = \text {ConvGRU}(\textbf{Z}_{i,t}, \textbf{H}_{t-1}^f), \quad \textbf{H}_t^b = \text {ConvGRU}(\textbf{Z}_{i,t}, \textbf{H}_{t+1}^b) \end{aligned}$$

Subsequently, the bidirectional information is aggregated to generate the final spatiotemporal representation $\textbf{Y}_i$, ensuring that both historical flow accumulation and future structural context contribute to the segmentation of the current frame:

3 $$\begin{aligned} \textbf{Y}_{i,t} = \tanh \left(\textbf{W}_f * \textbf{H}_t^f + \textbf{W}_b * \textbf{H}_t^b + \textbf{b}\right) \end{aligned}$$

where * denotes the convolution operation, and $\textbf{W}_f, \textbf{W}_b$ are learnable weights. By distributing this module across multiple encoder stages, the network maintains a continuous information pathway to the physically-enhanced hemodynamic data, effectively preventing the “wash-out” of micro-vascular structures in deep layers.

### Physically-guided spatiotemporal enhancement (PGSE)

Although the SFEM and BiConvGRU modules in the baseline architecture provide a mechanism for temporal feature integration, they operate as implicit learners that treat all pixel intensities with equal initial importance. This approach is often suboptimal for DSA sequences, where valid vascular signals are physically distinguishable from background noise through specific hemodynamic characteristics. To address this, we introduce the Physically-Guided Spatiotemporal Enhancement module at the network input stage. This module functions as a global pre-conditioner, calibrating the raw input sequence based on physical priors before it is processed by the encoder or injected into deep layers via SFEM.

The enhancement mechanism is constructed with two parallel branches targeting spatial and temporal domains, respectively. In the spatial domain, we leverage the observation that flowing contrast agents exhibit high intensity variance over time, whereas skeletal structures and background tissues remain relatively static. We compute the temporal standard deviation map of the input sequence to explicitly highlight active vascular regions. This variance map is then transformed into a spatial attention mask $\textbf{M}_{sp} \in \mathbb {R}^{1 \times H \times W}$ through a learnable *1 × 1* convolution followed by a Sigmoid activation. In the temporal domain, to prioritize the informative wavefront of the contrast bolus over residual wash-out signals, we impose a learnable time-decay vector $\textbf{W}_{tm} \in \mathbb {R}^{T}$. This vector assigns adaptive scalar weights to each frame, amplifying the contribution of phases with rich hemodynamic information.

Mathematically, let $\textbf{S} \in \mathbb {R}^{T \times C \times H \times W}$ denote the raw input sequence. The enhanced sequence $\hat{\textbf{S}}$ is obtained by modulating the raw input with both the spatial and temporal attention factors:

4 $$\begin{aligned} \textbf{M}_{sp} = \sigma \left( \mathscr {F}_{conv} \left( \sqrt{\frac{1}{T}\sum _{t=1}^{T}(\textbf{S}_t - \bar{\textbf{S}})^2} \right) \right) , \quad \hat{\textbf{S}}_{t} = \textbf{S}_{t} \otimes \textbf{W}_{tm, t} \otimes \textbf{M}_{sp} \end{aligned}$$

where $\mathscr {F}_{conv}$ denotes the *1 × 1* convolution, *σ* is the Sigmoid function, $\bar{\textbf{S}}$ represents the temporal mean of the input sequence, and *⊗* denotes the element-wise multiplication with implicit tensor broadcasting. Crucially, this enhancement propagates globally. By replacing the raw input $\textbf{S}$ with the enhanced $\hat{\textbf{S}}$ as the source for both the initial encoder layer and the multi-scale SFEM injections, we ensure that the entire network focuses on extracting features from topologically relevant vascular structures while effectively suppressing background artifacts.

### Wavelet-integrated feature encoding

Standard Convolutional Neural Networks typically rely on max-pooling or strided convolutions for downsampling. While efficient, these operations inevitably act as destructive filters, discarding high-frequency spatial details such as sharp vessel boundaries. To address this, we integrate discrete wavelet theory into the feature encoding backbone. Specifically, we employ a Haar Wavelet Downsampling (HWD) module^28^ to perform lossless resolution reduction, followed by Wavelet Transform Convolution (WTConv) layers^29^ for enhanced feature extraction.

The HWD module operates on the principle of lossless bijective mapping. The transformation is grounded in the orthogonal Haar basis formulation^28^. Let *ϕ (x)* denote the scaling function and *ψ (x)* denote the wavelet function. The 1-stage Haar transform decomposes a signal using the basis functions defined as:

5 $$\begin{aligned} \phi (x) = {\left\{ \begin{array}{ll} 1, & 0 \le x < 1 \\ 0, & \text {otherwise} \end{array}\right. }, \quad \left\{ \begin{aligned} \phi _1(x)&= \frac{1}{\sqrt{2}}\phi (2x) + \frac{1}{\sqrt{2}}\phi (2x-1) \\ \psi _1(x)&= \frac{1}{\sqrt{2}}\phi (2x) - \frac{1}{\sqrt{2}}\phi (2x-1) \end{aligned} \right. \end{aligned}$$

Equation 5 implies that a signal can be decomposed into approximation and detail components. Extending this to the 2D spatial domain of a feature map $\textbf{X} \in \mathbb {R}^{C \times H \times W}$, the transform generates four frequency sub-bands: the low-frequency approximation ($\textbf{X}_{LL}$) and three high-frequency detail components ($\textbf{X}_{LH}, \textbf{X}_{HL}, \textbf{X}_{HH}$). Unlike standard pooling, we concatenate these four components along the channel dimension, reducing the spatial resolution to $\frac{H}{2} \times \frac{W}{2}$ while quadrupling the channel dimension. Subsequently, a *1 × 1* convolution layer is applied to fuse these channels, ensuring that the full spectral integrity of the vascular structure is preserved.

**Figure 2:**
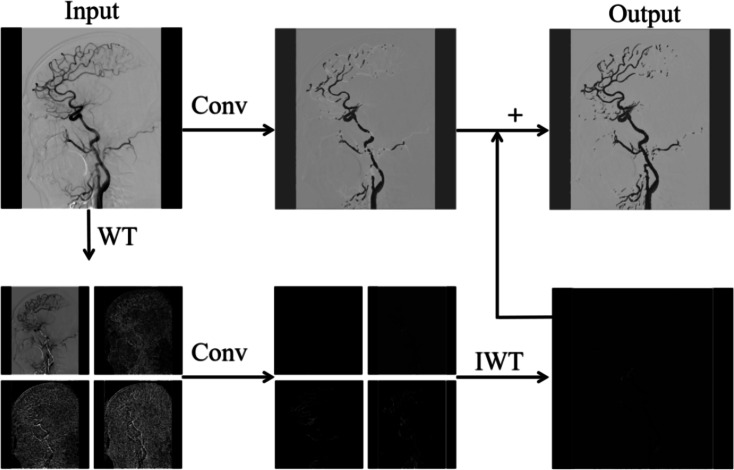
Visualization of the WTConv Module Workflow. This process expands the receptive field efficiently to capture global morphology.

Complementing the lossless downsampling, we introduce WTConv layers to enhance the receptive field, as illustrated in Fig. 2. Based on previous derivations^29^, the Haar Wavelet Transform (WT) can be formulated as a depth-wise convolution with stride 2 using a set of four orthogonal filters. Let $f_{LL}, f_{LH}, f_{HL}, f_{HH}$ denote the filters for the approximation and detail coefficients, respectively. They are defined as:

6 $$\begin{aligned} f_{LL} = \frac{1}{2}\begin{bmatrix} 1 & 1 \\ 1 & 1 \end{bmatrix}, \quad f_{LH} = \frac{1}{2}\begin{bmatrix} 1 & -1 \\ 1 & -1 \end{bmatrix}, \quad f_{HL} = \frac{1}{2}\begin{bmatrix} 1 & 1 \\ -1 & -1 \end{bmatrix}, \quad f_{HH} = \frac{1}{2}\begin{bmatrix} 1 & -1 \\ -1 & 1 \end{bmatrix} \end{aligned}$$

The WTConv layer leverages these filters to perform convolution within the wavelet domain. Formally, for an input tensor $\textbf{X}$ and a trainable weight tensor $\textbf{W}$, the operation is defined as a sequence of Wavelet Transform, convolution, and Inverse Wavelet Transform (IWT):

7 $$\begin{aligned} \textbf{Y} = \text {IWT}\left(\text {Conv}\left(\textbf{W}, \text {WT}(\textbf{X})\right)\right) \end{aligned}$$

Through this process, the WTConv module expands the convolution’s receptive field without dramatically increasing the number of parameters. This allows the network to better capture low-frequency information, which is beneficial for vascular structure recognition in brain vascular segmentation tasks.

To capture multi-scale dependencies, this process is applied recursively. Let $\textbf{X}^{(i)}_{LL}$ be the low-frequency input at level *i*. The network decomposes $\textbf{X}^{(i)}_{LL}$ into the next-level sub-bands, applies convolutions $\textbf{W}^{(i)}$ to each band, and aggregates the results via IWT. The recursive aggregation for a multi-level WTConv is given by:

8 $$\begin{aligned} \textbf{Z}^{(i)} = \text {IWT}\left(\textbf{Y}^{(i)}_{LL} + \textbf{Z}^{(i+1)}, \textbf{Y}^{(i)}_{H}\right) \end{aligned}$$

where $\textbf{Z}^{(i)}$ represents the aggregated output from level *i* onward. This cascade structure allows the network to utilize a small kernel size (e.g., *3 × 3*) to effectively respond to low frequencies corresponding to a receptive field that grows exponentially with the wavelet levels, which is important for capturing the global morphology of the arterial tree.

### Topology-aware reconstruction module

In the decoding trajectory, the primary objective is to recover the spatial resolution of the vascular probability map while maintaining the topological connectivity of fine vessels. Standard U-Net architectures typically rely on simple skip connection concatenation followed by bilinear upsampling or transposed convolutions. However, this naive approach suffers from two drawbacks: (1) the propagation of irrelevant background noise (e.g., skull artifacts) from the encoder, and (2) the blurring of vessel boundaries due to the limited receptive field of standard upsampling operators. To address these challenges, we propose a Topology-Aware Reconstruction strategy that integrates a Skip-Connection Gating mechanism with a Wavelet-Enhanced EUCB.

Before fusing the encoder features $\textbf{F}_{skip}$ with the decoder, we introduce a lightweight gating operation to suppress non-vascular signals. Unlike direct concatenation, this mechanism acts as a feature selector. We calculate a channel-wise attention vector $\textbf{w}$ using a global average pooling layer followed by a multi-layer perceptron. The original features are then re-calibrated:

9 $$\begin{aligned} \textbf{w} = \sigma \left(\textbf{W}_2 \delta \left(\textbf{W}_1 \text {AvgPool}(\textbf{F}_{skip})\right)\right), \quad \tilde{\textbf{F}}_{skip} = \textbf{F}_{skip} \cdot \textbf{w} \end{aligned}$$

where $\tilde{\textbf{F}}_{skip}$ denotes the filtered features. This ensures that the subsequent reconstruction module receives high-fidelity vascular information.

For the upsampling stage, we adopt the Efficient Upsampling Convolution Block (EUCB)^30^ as the backbone but introduce modifications to suit vascular topology recovery. The original EUCB relies on standard depth-wise convolutions (DWC) to capture spatial information, formulated as:

10 $$\begin{aligned} \textbf{Y}_{orig} = \mathscr {F}_{pwc}\left(\sigma \left(\text {BN}(\text {DWC}(Up(\textbf{X}_{in})))\right)\right) \end{aligned}$$

We identify that the standard DWC is insufficient for capturing multi-scale frequency details required for fine vessels. Therefore, we propose a *Wavelet-Enhanced EUCB*.

As described in the previous section, our block first applies nearest-neighbor interpolation *Up(· )* to scale the feature map. Crucially, we replace the standard DWC with the WTConv layer to refine the upscaled features in the frequency domain. To facilitate information exchange across feature channels, we append a channel shuffle operation $\mathscr {S}(\cdot )$ before the final pointwise convolution $\mathscr {F}_{pwc}$. The enhanced upsampling process is formulated as:

11 $$\begin{aligned} \textbf{Y}_{out} = \mathscr {F}_{pwc}\left(\mathscr {S}\left(\text {WTConv}(Up(\textbf{X}_{in}))\right)\right) \end{aligned}$$

By integrating WTConv, this enhanced block effectively mitigates aliasing artifacts and expands the receptive field during reconstruction. Finally, following the concatenation of the gated skip features and the upsampled maps, the fused features are further processed by a residual WTConv block. This design ensures that the multi-scale frequency analysis is consistently applied throughout the decoding trajectory, preserving the structural continuity of the restored vessels.

## Experiments

In this section, we conduct a series of experiments to evaluate the effectiveness of WESCP-Net. We first describe the experimental setup and evaluation metrics, followed by a comprehensive performance comparison with state-of-the-art methods, and finally perform an ablation study to analyze the contribution of each proposed module.

### Experimental setup

The proposed framework was implemented using Python 3.7 and PyTorch 1.12.1 on a workstation equipped with a single NVIDIA RTX 3090 GPU (24GB VRAM). We trained the model using the AdamW optimizer with a weight decay of 0.01 and a batch size of 64. The initial learning rate was set to $5 \times 10^{-4}$ and adjusted via a cosine annealing scheduler. The training was performed for a total of 200 epochs, with 100 iterations per epoch to ensure fairness in comparison.

We utilized the DIAS dataset, which was partitioned at the patient level into 30 training, 10 validation, and 20 test cases. Following the standard dataset protocols, all DSA sequences were strictly aligned to a temporal length of 8 frames, and instance-level Z-score normalization was applied.

Quantitative performance was evaluated using standard metrics including Dice Similarity Coefficient (DSC), Accuracy (Acc), Intersection over Union (IoU), and Area Under the Curve (AUC). Additionally, to assess the preservation of vascular topology, we utilized the clDice metric^23^. Unlike pixel-wise metrics, clDice evaluates structural continuity by measuring the overlap between the extracted vascular skeletons. Let $S_P$ and $S_L$ denote the skeletons extracted from the predicted mask ($V_P$) and the ground truth ($V_L$), respectively. clDice is defined as the harmonic mean of Topology Precision (*Tprec*) and Topology Sensitivity (*Tsens*):

12 $$\begin{aligned} & Tprec(S_P, V_L) = \frac{|S_P \cap V_L|}{|S_P|}, \quad Tsens(S_L, V_P) = \frac{|S_L \cap V_P|}{|S_L|}\end{aligned}$$

13 $$\begin{aligned} & clDice(V_P, V_L) = \frac{2 \cdot Tprec(S_P, V_L) \cdot Tsens(S_L, V_P)}{Tprec(S_P, V_L) + Tsens(S_L, V_P)} \end{aligned}$$

### Performance comparison

In this section, we present a comprehensive performance comparison of WESCP-Net against state-of-the-art methods across multiple evaluation metrics. Table 2 summarizes the quantitative results for representative 2D, 3D, and projection-based models, with the best results highlighted in bold.

**Table 2:** Performance comparison of different methods.

Type	Methods	DSC*↑*	Acc*↑*	IOU*↑*	AUC*↑*	clDice*↑*
2D	Res-UNet^9^	0.7494 ± 0.0604	0.9609 ± 0.0185	0.6030 ± 0.0774	0.9786 ± 0.0132	0.6748 ± 0.0858
	UNet++^8^	0.7569 ± 0.0544	0.9627 ± 0.0145	0.6120 ± 0.0712	0.9804 ± 0.0102	0.6799 ± 0.0080
	Att-UNet^31^	0.7605 ± 0.0548	0.9622 ± 0.0167	0.6167 ± 0.0707	0.9808 ± 0.0105	0.6873 ± 0.0759
	$\text {CS}^2$-Net^32^	0.7613 ± 0.0516	0.9637 ± 0.0132	0.6173 ± 0.0659	0.9814 ± 0.0099	0.6851 ± 0.0791
	nnU-Net^10^	0.7754 ± 0.0623	0.9665 ± 0.0107	0.6348 ± 0.0737	0.9799 ± 0.0121	0.6921 ± 0.0578
3D	UNet^2^	0.7450 ± 0.0587	0.9616 ± 0.0160	0.5972 ± 0.0759	0.9783 ± 0.0110	0.6709 ± 0.0841
	UNetR^15^	0.7517 ± 0.0584	0.9605 ± 0.0142	0.6064 ± 0.0743	0.9789 ± 0.0129	0.6737 ± 0.0716
	UNetR++^33^	0.7842 ± 0.0483	0.9643 ± 0.0135	0.6361 ± 0.0511	0.9833 ± 0.0094	0.7034 ± 0.0734
	Res-UNet^9^	0.7459 ± 0.0642	0.9634 ± 0.0114	0.5989 ± 0.0799	0.9775 ± 0.0146	0.6695 ± 0.0886
	UNet++^8^	0.7473 ± 0.0618	0.9632 ± 0.0121	0.6004 ± 0.0780	0.9789 ± 0.0119	0.6615 ± 0.0965
	Att-UNet^31^	0.7470 ± 0.0607	0.9606 ± 0.0166	0.5999 ± 0.0774	0.9787 ± 0.0113	0.6669 ± 0.0828
	FR-UNet^34^	0.7648 ± 0.0499	0.9648 ± 0.0120	0.6219 ± 0.0651	0.9816 ± 0.0092	0.6904 ± 0.0752
3D*→*2D	PSC^26^	0.7536 ± 0.0591	0.9640 ± 0.0115	0.6082 ± 0.0749	0.9788 ± 0.0130	0.6755 ± 0.0810
	SVS-Net^20^	0.7449 ± 0.0613	0.9629 ± 0.0140	0.5972 ± 0.0769	0.9766 ± 0.0132	0.6590 ± 0.0920
	VSS-Net^4^	0.7805 ± 0.0317	0.9667 ± 0.0109	0.6411 ± 0.0425	**0.9849** ± 0.0067	0.7073 ± 0.0438
	WESCP-Net	**0.7982** ± 0.0311	**0.9706** ± 0.0112	**0.6422** ± 0.0389	0.9796 ± 0.0173	**0.7135** ± 0.0523

WESCP-Net demonstrates favorable performance compared to pure 2D architectures. As illustrated in Table 2, standard 2D methods generally yield lower segmentation scores, primarily because they process frames independently and do not fully leverage the temporal information inherent in DSA sequences. For instance, UNet++ and Att-UNet achieve DSC scores of 0.7569 and 0.7605, respectively, whereas WESCP-Net achieves a higher DSC of 0.7982. Compared to the robust nnU-Net (DSC 0.7754), our method yields a measurable improvement. This performance difference suggests the effectiveness of our spatiotemporal modeling, which incorporates dynamic flow information that is typically unutilized in frame-by-frame 2D processing.

In the domain of 3D and sequence-based segmentation, WESCP-Net achieves competitive performance alongside traditional CNN-based models and recent Transformer-based architectures. Compared to the strong baseline VSS-Net, our method increases the DSC from 0.7805 to 0.7982 and maintains a comparable IoU (0.6422 vs. 0.6411). Notably, WESCP-Net also achieves a higher DSC than the Transformer-based UNetR++ (DSC 0.7842) and the video-based FR-UNet (DSC 0.7648). This suggests that while Transformers excel at capturing global context, the Wavelet-Integrated design of WESCP-Net can be highly suitable for preserving high-frequency spatial details, such as the boundaries of thin vessels, which are critical for accurate vascular segmentation.

The 1.8% numerical improvement in DSC over VSS-Net remains clinically relevant in the context of cerebral vascular segmentation. DSA images are characterized by severe class imbalance, where vessel pixels occupy a minimal fraction of the spatial domain. In such scenarios, a moderate gain in overlap metrics often corresponds to valuable corrections in difficult regions, specifically thin distal vessels. This is corroborated by the improvement in the connectivity-aware metric, clDice, which rises from 0.7073 (VSS-Net) to 0.7135 (WESCP-Net). To confirm the statistical significance of these improvements, a paired Wilcoxon signed-rank test was conducted across the 20 test subjects. The test demonstrated statistically significant gains over the VSS-Net baseline for both DSC (*p = 0.0021*) and clDice (*p = 0.0086*).

The higher clDice score suggests that our model goes beyond simple pixel-wise classification, indicating an enhanced ability to maintain the topological continuity of the vascular tree and reduce structural fractures. Despite the improvements in DSC and connectivity metrics, WESCP-Net yields a slightly lower AUC compared to the VSS-Net baseline, dropping from 0.9849 to 0.9796. We attribute this minor decrease to a potential trade-off associated with the network’s optimization for topological completeness. As a threshold-independent metric, AUC evaluates the continuous probability ranking, making it particularly sensitive to the massive background class prevalent in DSA images. To better preserve the connectivity of micro-vessels and achieve higher clDice scores, WESCP-Net is designed to maintain high sensitivity to faint distal vascular signals. This heightened sensitivity to weak topological features may also introduce minor probability fluctuations in background regions that share similar textures. These fluctuations can slightly disrupt the optimal probability ranking required for a higher AUC score. Upon binarization at the standard operational threshold, these structurally complete predictions tend to result in improved spatial overlap and better topological fidelity.

**Figure 3:**
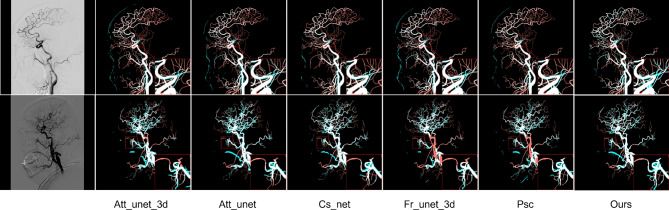
The segmentation details of the same case by different networks.

WESCP-Net achieves a higher Accuracy (0.9706) compared to the baseline (0.9667). This phenomenon can be attributed to the dominance of background pixels in the accuracy calculation. The concurrent improvement in DSC and Accuracy, alongside a stable IoU, suggests that the model is not simply overfitting the background, a scenario that would typically raise Accuracy but lower spatial overlap metrics. Instead, this behavior likely stems from the model’s enhanced ability to distinguish valid vascular signals from noise at the boundary regions. This precision is facilitated by the PGSE module, which suppresses background artifacts based on physical priors, and the Wavelet-Integrated encoder, which sharpens the feature representation of vessel edges. A qualitative comparison is provided in Fig. 3, where red arrows indicate regions where our method recovers vascular continuity that is often fragmented in other network predictions.

**Figure 4:**
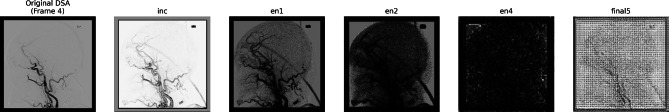
Visualization of internal feature maps of VSS-Net. The images display the progression from shallow layers (inc, en1) extracting texture details to deep layers (en4) capturing semantic topology. The final output (final5) yields the segmentation prediction. Darker regions represent higher activation.

To further investigate the underlying reasons for this performance gain and explore the internal feature extraction process, we visualized the intermediate feature maps of VSS-Net at varying depths, as shown in Fig. 4. For clarity, all feature maps are displayed in inverted grayscale, where darker pixels indicate higher neuronal activation levels. The visualization demonstrates distinct characteristics of hierarchical feature learning. The shallow layers, such as the inc and en1 modules, primarily function as edge detectors, preserving rich vessel textures and high-frequency spatial details. As the network depth increases, the intermediate layer (en2) begins to progressively suppress background noise. Features at the deep bottleneck (en4) become highly abstract; despite the reduced resolution, they effectively encode the global topological structure of the vessel tree. Ultimately, the output layer (final5) integrates these multi-scale features to generate the final pixel-level segmentation results.

### Ablation study

To evaluate the contribution of each proposed component, we conducted an ablation study. We decomposed the WESCP-Net into three core modules corresponding to the methodology sections: the Physically-Guided Spatiotemporal Enhancement, denoted as PGSE in Table 3; the Wavelet-Integrated Feature Encoding, denoted as WT-Enc; and the Topology-Aware Reconstruction module, denoted as TA-Dec. Table 3 details the incremental performance gains of these configurations compared to the VSS-Net baseline, with the best results highlighted in bold.

**Table 3:** Ablation study on the contribution of PGSE, topology-aware reconstruction (TA-Dec), and wavelet-integrated encoding (WT-Enc).

Config	PGSE	TA-Dec	WT-Enc	DSC*↑*	Acc*↑*	IOU*↑*	AUC*↑*	clDice*↑*
M0: VSS-Net				0.7805 ± 0.0317	0.9667 ± 0.0109	0.6411 ± 0.0425	**0.9849** ± 0.0067	0.7073 ± 0.0438
M1: Baseline + TA-Dec		*\checkmark*		0.7804 ± 0.0306	0.9673 ± 0.0111	0.6409 ± 0.0414	0.9845 ± 0.0066	0.7012 ± 0.0442
M2: Baseline + PGSE	*\checkmark*			0.7892 ± 0.0324	0.9693 ± 0.0132	0.6412 ± 0.0562	0.9825 ± 0.0106	0.7091 ± 0.0476
M3: PGSE + TA-Dec	*\checkmark*	*\checkmark*		0.7911 ± 0.0349	**0.9720** ± 0.0107	0.6419 ± 0.0383	0.9831 ± 0.0128	0.7112 ± 0.0583
M4: WESCP-Net	*\checkmark*	*\checkmark*	*\checkmark*	**0.7982** ± 0.0311	0.9706 ± 0.0112	**0.6422** ± 0.0389	0.9796 ± 0.0173	**0.7135** ± 0.0523

We first investigated the impact of the PGSE module (M2). By integrating physical priors to suppress background noise at the input stage, the model achieved a noticeable improvement in DSC (from 0.7805 to 0.7892) and Accuracy (from 0.9667 to 0.9693) compared to the baseline. This suggests that explicitly highlighting hemodynamic changes in the raw sequence provides cleaner features, helping to mitigate the network’s tendency to overfit to static skeletal artifacts.

Building upon the PGSE-enhanced features, we introduced the TA-Dec module (M3). This configuration yielded further incremental gains, increasing the DSC to 0.7911. Furthermore, the clDice metric rose to 0.7112. This improvement indicates that the proposed reconstruction strategy–incorporating Gating and Wavelet-Enhanced EUCB–helps bridge disconnected vascular segments that standard upsampling layers may struggle to recover.

Finally, the complete WESCP-Net (M4), which incorporates the WT-Enc module, achieved the highest DSC (0.7982) and clDice (0.7135) among the tested configurations. The wavelet transform allows the encoder to decompose features into frequency sub-bands, preserving high-frequency edge details during downsampling. This capability is highly beneficial for segmenting thin distal vessels, whose features are often attenuated in standard pooling operations.

It is worth noting the result of M1, where we applied the TA-Dec module without the PGSE or WT-Enc. The performance remained comparable to the baseline (DSC 0.7804), with a slight drop in clDice. This observation suggests a synergistic relationship: the advanced topology-aware decoder benefits from the high-fidelity features–provided by either the PGSE’s denoising or the Wavelet encoder’s detail preservation–to function effectively. The integration of these modules enables the network to achieve its enhanced segmentation accuracy and topological consistency.

## Discussion

In this study, we presented WESCP-Net, a specialized framework designed to address the challenges of cerebrovascular segmentation in spatiotemporal DSA sequences. The primary obstacles in this domain–specifically the loss of fine distal vessel details during downsampling and the interference of complex background noise–prompted the exploration of modules tailored for these data characteristics. Our results suggest that by integrating physical priors through the PGSE module and leveraging the frequency-preserving properties of wavelet transforms, WESCP-Net achieves competitive performance on the DIAS dataset, particularly in maintaining vascular topology.

Performance comparisons reveal characteristic differences between various paradigms when applied to angiographic data. Standard 2D methods, such as UNet++ and Att-UNet, yielded lower scores compared to spatiotemporal models. This behavior is largely attributable to the independent processing of DSA frames, which does not explicitly model the hemodynamic flow information that helps distinguish contrast-filled vessels from static bone artifacts. While sequence-based methods like VSS-Net improve upon this by incorporating temporal dependencies, they typically rely on standard convolutional downsampling layers, which can act as low-pass filters that attenuate fine details. Our experiments indicate that utilizing Haar Wavelet Downsampling facilitates the preservation of high-frequency spatial information. Notably, our method also showed improvements over Transformer-based architectures like UNetR++. While Transformers excel at modeling global semantic contexts, they may face limitations in capturing the fine-grained boundaries of distal vessels, a domain where Wavelet-Integrated encoding provides a valuable advantage.

The ablation study provides further insights into the mechanisms of the observed performance gains, suggesting that domain-specific data enhancement plays a key role alongside architectural design. We observed that the PGSE module, which serves as a physics-driven pre-processor, showed a notable contribution to the overall improvement. This aligns with the nature of DSA imaging: unlike standard MRI or CT, DSA results are inherently tied to the dynamic flow of contrast agents. By explicitly modeling this hemodynamic variance, the PGSE module facilitates data pre-conditioning before the sequence enters the deep network. These findings suggest that in specialized medical tasks, incorporating domain-specific physical priors, such as blood flow dynamics, is a valuable strategy that complements the deepening of neural network layers. Furthermore, the limited performance of the standalone Topology-Aware Decoder (Configuration M1) highlights a critical dependency: sophisticated reconstruction modules require high-fidelity feature representations–provided by the denoising effects of PGSE–to function effectively.

The relationship between the modest numerical improvement in DSC and the observed enhancement in vessel connectivity is worth noting. While cerebral vascular segmentation shares similarities with retinal vessel segmentation in terms of targeting thin, curvilinear structures, the DSA task presents distinct challenges due to lower contrast-to-noise ratios and skull interference. In such high-difficulty scenarios, the DSC metric can be influenced by the correct classification of background pixels and major arteries, which may limit its sensitivity to improvements in micro-vasculature. The higher clDice score achieved by WESCP-Net reflects a clinically relevant result. In interventional neuroradiology, the topological continuity of the vascular tree is essential; disconnected segments can mislead path-planning algorithms and affect catheter navigation. The ability of WESCP-Net to maintain structural integrity in distal branches holds clinical value that is not fully captured by pixel-level metrics alone.

This study has several limitations. First, the evaluation was conducted on a single-center dataset. While the data are representative of clinical stroke scenarios, multi-center validation is necessary to assess generalization across different angiographic equipment. Second, the current framework processes 2D+t sequences and does not reconstruct full 3D volumetric models, which remains a key requirement for surgical navigation. Future work may focus on extending the Wavelet-Based logic to 3D rotational angiography and exploring semi-supervised or unsupervised learning strategies to reduce the reliance on pixel-level manual annotations.

In conclusion, WESCP-Net presents a specialized approach for DSA segmentation. By modeling hemodynamic variations and utilizing frequency-preserving wavelet transforms, the proposed framework offers an effective method for segmenting cerebrovascular structures in clinical sequences.

## Conclusion

In this paper, we proposed WESCP-Net, a novel spatiotemporal segmentation framework designed to delineate intricate cerebral vasculature from DSA sequences. By synergizing the Physically-Guided Spatiotemporal Enhancement (PGSE) module with a Wavelet-Integrated encoder-decoder architecture, our method effectively resolves the conflict between noise suppression and high-frequency detail preservation. Extensive experiments demonstrate that WESCP-Net achieves state-of-the-art performance on the DIAS dataset. Beyond standard pixel-level accuracy, our approach improves topological connectivity, as evidenced by superior clDice scores. This study highlights the critical value of integrating physical hemodynamic priors and frequency-domain analysis into deep learning models, providing a robust tool for clinical diagnosis and interventional planning. Future work will extend this paradigm to 3D volumetric reconstruction to further enhance surgical navigation capabilities.

## Data Availability

The dataset used in this study is the DIAS, which is publicly available. All data included in this study are available upon request by contact with the corresponding author.

## Code availability

The custom code used to support the findings of this study has been deposited in the Zenodo repository under the DOI: 10.5281/zenodo.19688209 with restricted access. The code is available upon reasonable request to the corresponding author for research purposes. Requesters are kindly asked to include their institutional affiliation and a brief description of the intended purpose in their request.
